# Postural and biomechanical insights in disc displacement with reduction

**DOI:** 10.22514/jofph.2025.030

**Published:** 2025-06-12

**Authors:** Ömer Dursun, Abdurrahman Tanhan, Mesut Arslan, Rabia Çelikel

**Affiliations:** ^1^Department of Physiotherapy and Rehabilitation, Faculty of Health Sciences, Bitlis Eren University, 13100 Bitlis, Turkey

**Keywords:** Posture, Temporomandibular joint disorders, Pain threshold

## Abstract

**Background**: Although the relationship between temporomandibular disorders and posture has been addressed in many studies, a complete consensus has not yet been reached. This study aimed to evaluate global posture, cervical, thoracic and lumbar mobility, and the biomechanical characteristics of muscles in individuals with disc displacement with reduction. **Methods**: A total of 70 participants were included in the study, consisting of 37 healthy individuals in the control group and 33 individuals in the study group. The participants included in the study were assessed for temporomandibular joint range of motion, pressure pain threshold in regions related to the temporomandibular disorders (lateral capsule, masticatory muscles and upper trapezius), global posture and thoracic and lumbar spine mobility, cervical posture and mobility, and the biomechanical characteristics (elasticity, stiffness and tone) of the paravertebral muscles, masticatory muscles and upper trapezius. The assessment was conducted using a digital caliper, an analog algometer, a spinal mouse, a universal and modified universal goniometer, and MyotonPRO, respectively. **Results**: Cervical and temporomandibular joint range of motion were similar in both groups. The study group showed lower pressure pain threshold overall, except in the left upper trapezius and right anterior temporalis. The biomechanical characteristics (elasticity, stiffness and tone) of the masseter muscle and paravertebral muscles were also comparable, except for reduced elasticity in the left semispinalis muscle. Global posture and mobility (cervical, thoracic and lumbar) were alike in both groups. **Conclusions**: This study showed that young adults with disc displacement with reduction largely have similar global posture, mobility (cervical, thoracic and lumbar) and muscle biomechanical characteristics (elasticity, stiffness and tone) compared to healthy individuals. However, their pressure pain threshold, especially at the joint capsule and most measurement points, was lower, except for a few areas.

## 1. Introduction

The temporomandibular joint (TMJ), as a component of the stomatognathic system, plays an essential role in a variety of vital functions, including mastication and swallowing [1, 2]. Due to the complex nature of these functions and the high level of coordination required to perform them [1], the TMJ works in harmony with another system, the craniocervical system, through its anatomical, biomechanical and neurological connections [3]. This close relationship, necessary for the proper functioning of the TMJ, makes the joint susceptible to dysfunctions occurring in the craniocervical system [3]. If the adaptive capacity of the TMJ is exceeded, temporomandibular disorders (TMD) may develop [4]. Postural disorders of the cervical spine, especially forward head posture, are among the most emphasized issues in the development of TMD, and considerable focus is placed on head posture in this context [5].

Forward head posture characterized by upper cervical extension and lower cervical flexion [6] induces muscular and articular adaptations in the TMJ. Forward head posture results in increased masticatory muscle activity, and posterior migration of the mandibular condyle [6]. Change in the biomechanic of the TMJ due to forward head posture causes increased compression in the bilaminar zone of the disc [6] and could gradually induce pathological changes characterized by inflammation and masticatory muscle spasms [7] and might set the pathway for the development of TMD [4]. In addition, forward head posture disrupts the condyle-disc relationship by the posterior migration of the mandibular condyle [6]. Considering the fact that more than half of the individuals with internal derangement (52.5%) have posteriorly displaced condyle [8], forward head posture which result in aforementioned disruption [6] might be associated with the development of disc displacement with reduction (DDwR) [8]. One of the causes of forward head posture is inflammation [7], which leads to the production of synovial fluid characterized by reduced lubrication capacity and increased friction. This condition increases shear stress on joint surfaces, the disc and articular cartilage, potentially triggering the development of internal derangement [4]. The changes caused by forward head posture in the TMJ [6] have laid the groundwork for investigating this postural disorder in TMD [5]. However, assessment of postural disorders in TMD cannot be merely restricted to the cervical spine [9, 10].

The relationship between the cervical spine and the TMJ is also present in a similar way with the cervical and thoracic spine [11]. Upper, middle and lower segments of the thoracic spine (*i.e.*, T1, T6 and T12) contribute to all movements of the cervical spine [11]. This relationship between cervical and thoracic spine is emphasized by the term “regional interdependence” by Wainner *et al*. [12]. Apart from this indirect evidence showing the fact that TMD might induce global postural changes, there is direct proof on this issue as well [9, 10, 13].

Although there are studies addressing both cervical and global posture in TMD [1], the lack of consensus on the relationship between TMD and cervical spine [5], and global body posture [14] along with the lack of high-quality studies and the relatively limited number of studies focusing on global posture [14], necessitates a more comprehensive and integrative evaluation of the topic, especially for the DDwR. Although the relationship between myogenic TMD and postural disorders is established on solid grounds it is hard to say the same for the DDwR [3]. The heterogeneity in methodologies and study designs of existing research raises questions about the reliability of findings regarding both head posture and global posture assessments in individuals with DDwR, making it difficult to draw general conclusions from the results [5].

From this perspective, this study aims to evaluate global posture and muscular adaptations in individuals with DDwR. Specifically, it seeks to answer the question: How do global posture and muscular adaptations change in individuals with DDwR?

## 2. Materials and methods

This study was conducted at Bitlis Eren University, Department of Physiotherapy and Rehabilitation between April 2024 and July 2024. The study protocol was approved by the Non-Interventional Ethics Committee of Bitlis Eren University (Approval date: 15 March 2024, approval number: 2024/2-1). Prior to enrollment, participants were provided with verbal information about the study objectives and procedures, and their written consent was obtained. The sample size for the study was determined using a priori power analysis based on the craniovertebral angle values of the study of Yuan *et al*. [15]. According to power analysis performed with the average craniovertebral angle of both sexes (study group: 44.27° ± 5.86°, control group: 49.5° ± 5.01°) effect size was found to be 0.95. Due to large effect size of the study of Yuan *et al*. [15] priori power analysis with a large effect size (*d* = 0.80) and 80% power was performed. The power analysis indicated that each group should include 26 participants. It was determined that at least 29 participants should be included in each group, taking into account a 10% drop out. The study included individuals aged 18–30 without TMD symptoms as the control group, and individuals aged 18–30 with DDwR, diagnosed solely according to Diagnostic Criteria for Temporomandibular Disorders (DC/TMD) Axis I [16], who are not currently undergoing any treatment and previously not received any treatment as the study group. Individuals with missing teeth [17], neurological, metabolic, systemic or rheumatologic diseases, a history of musculoskeletal surgery within the last 3 months, a history of pregnancy, or mental health issues were excluded from the study. A total of 10 participants were excluded from the study based on inclusion and exclusion criteria, with five from the control group and five from the study group (Fig. 1).

**Fig. 1.** Study flow chart.

The participants included in the study were assessed for TMJ range of motion, pressure pain threshold in regions related to the TMD (lateral capsule, masticatory muscles and upper trapezius), global posture and thoracic and lumbar spine mobility, cervical posture and mobility, and the biomechanical characteristics (elasticity, stiffness and tone) of the paravertebral muscles, masticatory muscles and upper trapezius. TMJ and cervical range of motion, as well as cervical mobility, were assessed by researcher MA. The pressure pain threshold was measured by researcher AT, while global posture and mobility, along with the biomechanical characteristics of the muscles, were evaluated by researcher RÇ.

Participants’ TMJ range of motion was assessed using a digital caliper. Measurements were taken while the participants were seated in a long sitting position with their heads supported. For the mandibular depression movement, participants were asked to open their mouths as wide as possible. For mandibular lateral deviation, they were asked to move their jaws as far as possible to the side being measured, and for mandibular protrusion, participants were instructed to move their jaws forward as far as they can. For mandibular depression, the vertical distance between the central incisors was measured, and for mandibular protrusion, the horizontal distance between the central incisors were recorded. For mandibular lateral deviation, the position of the upper central incisor relative to the lower central incisor was marked using biocompatible markers before the movement. After the mandibular lateral deviation, the measurement was repeated and the horizontal distance between the two positions was measured [18].

For the pressure pain threshold assessment, participants’ masticatory muscles, TMJ lateral capsules, and trapezius muscles were bilaterally evaluated using a handheld analog algometer (Baseline, Fabrication Enterprises Inc., NY, USA). The pressure pain threshold for the masticatory muscles was measured at the most prominent point of the masseter muscle. For temporalis muscle, measurements were taken 2 cm posterior to its anterior border [19]. The lateral capsule measurement was performed at the lateral aspect of the joint, identified through palpation [20], and the upper trapezius pain threshold was measured at the midpoint between the acromion and the C7 spinous process [21] (Fig. 2). Three measurements were taken at each point, with a 30-second interval between each measurement, and the average value was recorded [22].

**Fig. 2.** Pressure pain threshold measurement points.

To assess cervical posture and mobility participants’ craniovertebral angle and cervical range of motion were measured. Craniovertebral angle was measured with modified universal goniometer while participants were standing. After the participants assumed the measurement position, the angle between the line connecting the C7 spinous process and the tragus of the ear, and the horizontal line passing through the C7 spinous process was measured using a goniometer. This method is reported to have good intra- and interrater reliability, with intraclass correlation coefficients (ICC) of 0.887 and 0.893, respectively [23]. Cervical range of motion was measured using a 10-inch manual goniometer while participants were seated in a relaxed posture. For cervical flexion and extension, the goniometer’s pivot was placed at the external auditory meatus, with the fixed arm vertical and the moving arm aligned with the base of the nostrils. Cervical rotation was measured from behind, positioning the pivot at the vertex, the fixed arm parallel to the acromion, and the moving arm aligned with the nose. Lateral flexion was also measured from behind, using the C7 spinous process as the pivot, the fixed arm parallel to the ground, and the moving arm following the midline of the cervical spine. Each measurement was repeated three times, and the average value was recorded [24].

The participants’ global posture and mobility in the sagittal plane were assessed using the Spinal Mouse (Spinal Mouse®, Idiag AG, Fehraltorf, ZH, Switzerland). With the aid of the device, the thoracic and lumbar spinal curvatures in the neutral position, as well as the total thoracic and lumbar mobility obtained from maximum flexion and maximum extension movements, were measured. Prior to the measurement, the measurement positions were demonstrated to the participants. Then, the spinous processes of C7 and S3 were marked on each participant. After the participants assumed the measurement positions, the device was slowly moved from cranial to caudal to record the measurements. The thoracic (T1–T12) and lumbar (L1–S1) spinal curvatures, sagittal inclination (the angle between the vertical line and the line connecting C7 and the sacrum), and thoracic and lumbar range of motion (the difference between maximum flexion and maximum extension) were calculated using the device’s software [25, 26].

The biomechanical characteristics (elasticity, stiffness and tone) of the participants’ paravertebral muscles, masticatory muscles and upper trapezius were assessed using a non-invasive device called MyotonPRO (MyotonPRO, Myoton AS, Tallinn, Estonia) [27]. The trapezius muscle measurement was performed with participants in a prone position, their hands hanging off the treatment table. The measurement was taken at the midpoint between the C7 spinous process and the acromion [21]. The device was positioned perpendicular to the measurement point, and three consecutive measurements were taken [28]. For the masseter muscle, measurements were taken with participants in a supine position [29], and their mandibles in a physiological rest position [30], defined as a 2–3 mm space between the incisors. The measurement point was marked bilaterally at the most prominent point of the masseter during maximum bite force, and five consecutive measurements were taken from this point [30]. The measurement of the extensor muscles at the C4 level (semispinalis capitis) was performed while participants are comfortably in a prone position. Measurements were taken 2 cm lateral to the C4 spinous process, with the device positioned perpendicular to the measurement point, and two consecutive measurements were obtained [31]. For the sternocleidomastoid muscle, measurements were taken while participants are in a supine position with their heads supported by a soft pillow. The measurement was made at the midpoint between the sternum and the mastoid process, and each measurement was repeated three times [32]. For the lumbar erector spinae muscles, the superior border of the iliac crest was first identified to determine the intervertebral space between the third and fourth lumbar vertebrae [33]. For lower lumbar muscle tone, L4 was used, for the upper lumbar region L1, and for the thoracic region, T1, T6 and T12 spinous processes were identified. The most prominent point of the erector spinae muscles adjacent to these spinous processes were determined and used as the measurement point. To identify the most prominent points, participants were asked to lift their heads and feet simultaneously [34]. The measurements were taken while participants are in a prone position, with their arms relaxed at their sides. To minimize the effect of intra-abdominal pressure on the measurements, participants were instructed to hold their breath for 5 seconds at the end of exhalation, during which time the measurements were performed. Three consecutive measurements were taken at each region [35].

Statistical analysis was performed using the SPSS 27 program (IBM, New York, NY, USA). Continuous variables were illustrated as average, standard deviation, and categorical variables as number and percentage. The normal distribution of the data was evaluated with the Shapiro-Wilk test. Whether or not the parametric assumptions were achieved, the independent sample *t*-test or Mann-Whitney U test was used for the intergroup comparison. Differences between the categorical variables were analyzed with the chi-square test. Correlation analysis was conducted using the Spearman’s rank correlation method. The statistical significance grade was determined as *p* < 0.05.

## 3. Results

Both groups’ physical characteristics and demographic data were homogeneous. More than half of the individuals had bilateral disc displacement, while a smaller proportion had mixed-type TMD (Table 1).

**Table 1.** Intergroup comparison of physical and sociodemographic characteristics and TMD characteristics.

		Control group (n = 37)		Study group (n = 33)					
		Mean ± s.d.	Median (min–max)	Mean ± s.d.	Median (min–max)	*t*	U	χ^2^	*p*
Age (yr)		-	21 (18–28)	-	20 (18–26)	N.A.	0.276	N.A.	0.783^b^
Height (cm)		169.67 ± 9.01	-	170.30 ± 9.61	-	0.282	N.A.	N.A.	0.779
Body weight (kg)		60 (46–87)	-	60 (44–90)	-	N.A.	0.353	N.A.	0.724^b^
BMI (kg/cm^2^)			21.7 (17.7–29.10)		21 (17.6–31.20)	N.A.	0.124	N.A.	0.902^b^
		n	%	n	%				
Gender									
	Male	17	46	13	39	N.A.	N.A.	0.306	0.580^a^
	Female	20	54	20	61				
DDwR side									
	Unilateral	-	-	18	49	N.A.	N.A.	N.A.	N.A.
	Bilateral	-	-	19	51				
TMD subtype									
	DDwR	-	-	29	88	N.A.	N.A.	N.A.	N.A.
	Mixed	-	-	4	12				

*p < 0.05 statistical significance independent t test; ^a^Chi-Square Test; ^b^Mann Whitney U test; N.A.: non-applicable; TMD: Temporomandibular disorders; DDwR: disc displacement with reduction; s.d.: standard deviation; BMI: body mass index; min: minimum; max: maximum.*

The cervical and TMJ range of motion were similar in both groups. The pressure pain threshold of the study group was significantly lower, except for the left upper trapezius and the right anterior temporalis (Table 2).

**Table 2.** Intergroup comparison of the pressure pain threshold, mandibular range of motion and cervical posture.

	Control group (n = 37)		Study group (n = 33)				
	mean ± s.d.	Median (min–max)	mean ± s.d.	Median (min–max)	*t*	U	*p*
Depression (mm)	47.72 ± 7.05	-	46.00 ± 7.49	-	0.995	N.A.	0.323
Protrusion (mm)	6.01 ± 2.27	-	6.07 ± 2.42	-	0.110	N.A.	0.913
Right lateral deviation (mm)	8.08 ± 2.75	-	8.30 ± 2.03	-	0.379	N.A.	0.706
Left lateral deviation (mm)	-	9.00 (4.00–12.00)	-	8.00 (4.00–12.00)	N.A.	0.308	0.758^a^
Cervical flexion (º)	59.51 ± 12.2	-	58.60 ± 10.8	-	0.328	N.A.	0.744
Cervical extension (º)	50.78 ± 9.23	-	51.84 ± 11.37	-	0.432	N.A.	0.667
Right cervical lateral flexion (º)	-	45.00 (25.00–58.00)	-	41.00 (28.00–58.00)	N.A.	1.823	0.068^a^
Left cervical lateral flexion (º)	41.40 ± 7.92	-	41.75 ± 8.31	-	0.181	N.A.	0.857
Craniovertebral angle (º)	40.24 ± 4.96	-	40.66 ± 7.14	-	0.285	N.A.	0.777
Right masseter PPT (kg/cm^2^)	-	2.75 (2.00–5.50)	-	2.55 (1.35–3.4)	N.A.	2.867	0.004*^a^
Left masseter PPT (kg/cm^2^)	-	2.70 (1.55–5.30)	-	2.35 (1.20–3.33)	N.A.	2.319	0.02*^a^
Right temporalis PPT (kg/cm^2^)	-	4.20 (2.50–8.00)	-	3.70 (2.50–6.35)	N.A.	1.318	0.187^a^
Left temporalis PPT (kg/cm^2^)	4.29 ± 1.13	-	3.75 ± 0.99	-	2.118	N.A.	0.038*
Right lateral capsule PPT (kg/cm^2^)	-	3.35 (2.30–6.50)	-	2.80 (1.45–3.90)	N.A.	3.013	0.003*^a^
Left lateral capsule PPT (kg/cm^2^)	3.38 ± 0.68	-	2.77 ± 0.80	-	3.415	N.A.	0.001*
Right upper trapezius PPT (kg/cm^2^)	-	4.55 (2.60–9.00)	-	3.60 (1.80–8.25)	N.A.	2.289	0.022*^a^
Left upper trapezius PPT (kg/cm^2^)	-	4.40 (2.40–9.00)	-	3.80 (2.00–8.05)	N.A.	1.383	0.167^a^

*p < 0.05 statistical significance, independent t test; ^a^Mann Whitney U test; PPT: pressure pain threshold; N.A.: non-applicable; s.d.: standard deviation; min: minimum; max: maximum.

The biomechanical characteristics of the masticatory muscles were similar in both groups. Additionally, the paravertebral biomechanical characteristics were comparable in both groups, except for the elasticity of the left semispinalis muscle (Table 3).

**Table 3.** Intergroup comparison of muscles’ biomechanical properties.

	Control group (n = 37)		Study group (n = 33)				
	Mean ± s.d.	Median (min–max)	Mean ± s.d.	Median (min–max)	*t*	U	*p*
Right masseter tone (Hz)	15.54 ± 1.56	-	15.45 ± 1.73	-	0.211	N.A.	0.834
Right masseter stiffness (N/m)	322.48 ± 66.12	-	329.15 ± 71.41	-	0.405	N.A.	0.686
Right masseter elasticity (log)	1.71 ± 0.15	-	1.70 ± 0.30	-	0.197	N.A.	0.845
Left masseter tone (Hz)	15.26 ± 1.67	-	15.11 ± 1.49	-	0.393	N.A.	0.696
Left masseter stiffness (N/m)	-	303 (217–589)	-	309 (219–409)	N.A.	0.065	0.948
Left masseter elasticity (log)	1.69 ± 0.17	-	1.68 ± 0.23	-	0.281	N.A.	0.780
Right semispinalis tone (Hz)	14.86 ± 1.88	-	15.18 ± 2.58	-	0.601	N.A.	0.550
Right semispinalis stiffness (N/m)	-	258 (139–409)	-	246 (134–570)	N.A.	0.159	0.874^a^
Right semispinalis elasticity (log)	-	1.14 (0.81–1.44)	-	1.12 (0.81–2.97)	N.A.	0.053	0.958^a^
Left semispinalis tone (Hz)	14.72 ± 1.76	-	14.96 ± 2.43	-	0.469	N.A.	0.641
Left semispinalis stiffness (N/m)	-	243 (148–414)	-	248 (138–637)		0.559	0.576^a^
Left semispinalis elasticity (log)	1.12 ± 0.14	-	1.04 ± 0.13	-	2.235	N.A.	0.028*
Right SCM tone (Hz)	-	13.0 (11.7–16.9)	-	13.1 (11.6–16.1)	N.A.	0.353	0.724^a^
Right SCM stiffness (N/m)	-	193 (156–342)	-	203 (145–353)	N.A.	0.983	0.326^a^
Right SCM elasticity (log)	-	1.07 (0.85–1.78)	-	1.06 (0.83–1.80)	N.A.	0.182	0.855^a^
Left SCM tone (Hz)	-	12.6 (11.0–16.5)	-	12.9 (11.5–16.2)	N.A.	1.207	0.227^a^
Left SCM stiffness (N/m)	-	185 (134–287)	-	197 (144–300)	N.A.	1.406	0.160^a^
Left SCM elasticity (log)	-	1.07 (0.89–1.69)	-	1.05 (0.81–1.53)	N.A.	0.336	0.737^a^
Right T1 tone (Hz)	14.87 ± 2.01	-	14.90 ± 2.02	-	0.080	N.A.	0.936
Right T1 stiffness (N/m)	-	276 (148–464)	-	276 (178–614)	N.A.	0.129	0.897^a^
Right T1 elasticity (log)	1.11 ± 0.17	-	1.14 ± 0.18	-	0.569	N.A.	0.572
Left T1 tone (Hz)	14.76 ± 2.17	-	14.75 ± 1.90	-	0.025	N.A.	0.980
Left T1 stiffness (N/m)	-	276 (157–493)	-	247 (165–498)	N.A.	0.024	0.981^a^
Left T1 elasticity (log)	-	1.17 (0.78–1.47)	-	1.14 (0.32–1.55)	N.A.	0.335	0.737^a^
Right T6 tone (Hz)	-	17.90 (11.10–21.40)	-	18.70 (13.70–21.30)	N.A.	1.448	0.148^a^
Right T6 stiffness (N/m)	363.10 ± 51.11	-	381.63 ± 59.65	-	1.399	N.A.	0.166
Right T6 elasticity (log)	1.04 ± 0.16	-	1.05 ± 0.14	-	0.233	N.A.	0.817
Left T6 tone (Hz)	-	18.04 (15.10–22.40)	-	18.60 (13.20–22.10)	N.A.	0.894	0.371^a^
Left T6 stiffness (N/m)	-	359 (278–527)	-	383 (236–517)	N.A.	0.847	0.397^a^
Left T6 elasticity (log)	1.07 ± 0.17	-	1.06 ± 0.12	-	0.393	N.A.	0.696
Right T12 tone (Hz)	-	14.40 (11.20–22.10)	-	14.00 (11.20–23.90)	N.A.	0.076	0.939^a^
Right T12 stiffness (N/m)	-	252 (145–593)	-	257 (156–727)	N.A.	0.088	0.930^a^
Right T12 elasticity (log)	-	1.07 (0.76–1.89)	-	1.12 (0.75–1.73)	N.A.	0.147	0.883^a^
Left T12 tone (Hz)	-	14.40 (11.40–19.80)	-	14.30 (10.80–24.50)	N.A.	0.229	0.818^a^
Left T12 stiffness (N/m)	-	237 (138–519)	-	236 (137–741)	N.A.	0.035	0.972^a^
Left T12 elasticity (log)	-	1.09 (0.67–1.68)	-	1.10 (0.70–1.86)	N.A.	0.606	0.544^a^
Right L1 tone (Hz)	-	13.6 (10.00–21.30)	-	13.2 (10.10–23.50)	N.A.	0.182	0.855^a^
Right L1 stiffness (N/m)	-	219 (113–547)	-	205 (117–715)	N.A.	0.453	0.651^a^
Right L1 elasticity (log)	-	1.02 (0.55–1.90)	-	1.05 (0.55–1.74)	N.A.	0.535	0.592^a^
Left L1 tone (Hz)	-	13.6 (10.40–21.00)	-	13.4 (10.30–23.8)	N.A.	0.736	0.462^a^
Left L1 stiffness (N/m)	-	205 (120–575)	-	207 (125–753)	N.A.	0.788	0.430^a^
Left L1 elasticity (log)	-	1.01 (0.54–1.41)	-	0.90 (0.59–1.65)	N.A.	0.135	0.892^a^
Right L4 tone (Hz)	-	13.30 (2.10–19.50)	-	12.50 (10.40–21.30)	N.A.	0.235	0.814^a^
Right L4 stiffness (N/m)	-	205 (130–485)	-	198 (127–635)	N.A.	0.571	0.568^a^
Right L4 elasticity (log)	-	0.91 (0.62–1.71)	-	0.93 (0.53–1.79)	N.A.	0.441	0.659^a^
Left L4 tone (Hz)	-	13.20 (9.90–17.80)	-	12.80 (10.30–20.80)	N.A.	0.235	0.814^a^
Left L4 stiffness (N/m)	-	200 (138–421)	-	193 (130–608)	N.A.	0.200	0.841^a^
Left L4 elasticity (log)	-	0.83 (0.61–1.85)	-	0.93 (0.50–2.00)	N.A.	1.771	0.077^a^

*p < 0.05 statistical significance independent t test; ^a^Mann Whitney U test; N.A.: non-applicable; s.d.: standard deviation; min: minimum; max: maximum; SCM: sternocleidomastoid muscle.

Global posture and mobility values were also similar in both groups (Table 4).

**Table 4.** Intergroup comparison of global spine mobility.

	Control group (n = 37)		Study group (n = 33)				
	Mean ± s.d.	Median (min–max)	Mean ± s.d.	Median (min–max)	*t*	U	*p*
Sagittal Thoracic Posture	37.91 ± 9.04	-	40.06 ± 9.07	-	0.988	N.A.	0.327
Sagittal Lumbar Posture	-	−30.24 (−60–24)	-	−33 (−59–12)	N.A.	0.865	0.387^a^
Sagittal Thoracic Mobility	-	23 (−39–45)	-	24 (4–60)	N.A.	0.212	0.832^a^
Sagittal Lumbar Mobility	-	74 (48–99)	-	76 (49–95)	N.A.	0.082	0.934^a^

*p < 0.05 statistical significance independent t test, ^a^Mann Whitney U test; N.A.: non-applicable; s.d.: standard deviation; min: minimum; max: maximum.*

No correlation between pain threshold measurement values and DDwR side (Table 5).

**Table 5.** Correlation between pressure pain threshold values and DDwR side.

	Right temporalis		Left temporalis		Right temporalis		Left temporalis		Right lateral capsule		Left lateral capsule		Right trapezius		Left trapezius	
	*r*	*p*	*r*	*p*	*r*	*p*	*r*	*p*	*r*	*p*	*r*	*p*	*r*	*p*	*r*	*p*
DDwR side	−0.137	0.448	0.085	0.639	−0.253	0.155	−0.147	0.415	−0.171	0.340	−0.184	0.305	−0.150	0.404	−0.206	0.249

*Spearman rank’s correlation. DDwR was categorized as bilateral and unilateral. DDwR: Disc displacement with reduction.*

## 4. Discussion

This study demonstrated that young adults with DDwR exhibit global posture and mobility, as well as muscle biomechanical characteristics, that are similar to those of healthy individuals, aside from random differences. Additionally, the study revealed that pressure pain threshold, particularly at the joint capsule and in most other measurement points except for a few, was reduced in young adults with DDwR.

Owing to the close interrelationship between TMJ and the craniocervical system, there are a pile of studies on the issue dating back to 1970s [1]. Despite more than half a century having passed [1] and a chronological shift towards more objective assessment methods over time, from visual posture evaluation to photographic measurement, radiographic imaging, stabilometry and rasterstereography [3, 5, 14, 36] the relationship between TMD and head posture has yet to be fully elucidated. On one side of the scale, studies point out that forward head posture is a postural irregularity seen in individuals with TMD [17, 37, 38] and on the other side studies emphasize the opposite [39, 40]. At this point it is hard to say which side is heavier than the other. In this study aiming to help elucidate which of the scale is heavier than the other by assessing the global posture characteristics of the individuals with DDwR, it was found that these individuals have similar global posture characteristics compared with healthy individuals.

In our study, the finding that individuals with DDwR share similar craniocervical postural characteristics with healthy individuals suggests that the diagnosis of TMD alone is not sufficient for the development of craniocervical postural disturbances. It appears that the symptom(s) accompanying TMD, or the collective impact of these symptoms in the form of jaw disability, must possess specific characteristics that trigger craniocervical postural changes in individuals with TMD [37]. Wiesinger *et al*. [41] describe the impact of the clinical features of TMD on craniocervical region symptoms using a “dose-response model” in their study. This model emphasizes a linear relationship between the severity and frequency of TMD symptoms and the symptoms in the craniocervical region. The combined impact of TMD symptoms, referred to as jaw disability, shows a strong correlation with neck disability [37, 42]. Additionally, there is a moderate positive correlation between TMD severity and neck disability, as well as a negative correlation between TMD severity and cervical muscle strength. Furthermore, the cervical muscle strength of individuals with TMD is significantly lower compared to healthy individuals [43]. Another study highlights a significant relationship between pain, TMD and head posture [44]. As can be inferred, for craniocervical postural changes to occur in individuals with TMD, the symptoms or cumulative impact of the disorder must surpass a threshold that has yet to be precisely identified [37]. In this context, the most emphasized symptom of TMD is pain and its characteristics [37, 45], which are closely associated with jaw disability and neck disability [45]. Pain, as it triggers “pain adaptation models” [37] characterized by muscle inhibition, excitation, or delayed activation [46], is frequently highlighted in studies either directly or indirectly as a criterion for including individuals with TMD. Pain characteristics, such as its intensity or referral pattern, have been particularly emphasized in this context by directly (moderate or severe pain or existence of pain) [37, 38, 47] or indirectly (in need of treatment) [18] determining a specific inclusion or exclusion criteria or a causal finding of the study [44]. Apart from the pain characteristic another emphasized one is the severity of the TMD [43]. Although these two factors have been emphasized in numerous studies, there are also a considerable number of studies in which these factors have been overlooked. This includes cases where individuals without need of treatment were included, pain or pain severity was not assessed, or these factors were not established as criteria [39, 40]. Interestingly, in studies that consider even one of the two aforementioned criteria, significant differences in the cervical spine posture or alignment of individuals with TMD are observed, regardless of the type of disorder [5]. Conversely, in studies where these criteria are not included, the opposite findings are reported [39, 40]. The undeniable impact of pain characteristics and the severity of TMD on craniocervical posture may have been overlooked in our study, potentially leading to both groups having similar craniovertebral angle values. Although we did not assess pain or its severity using surveys or scales such as the Visual Analog Scale (VAS) or the Numeric Pain Rating Scale, the fact that the pain threshold values of the study group exceeded the threshold defined for myofascial pain syndrome [48] and that the study group consisted of cases not in need of treatment indirectly indicates that the pain severity in the cases was below 30 mm. As is well known, a pain level below 30 mm on the VAS indicates that the individual does not need treatment [49]. This suggests that pain was either absent or of an intensity low enough not to require treatment in the study group. Given the relationship between pain severity and head posture [44], the low pain intensity might have prevented pain from exerting a pathological influence on craniocervical posture.

The number of studies evaluating global posture and mobility in TMD [9, 10, 39, 46] is fewer compared to those assessing cervical spine posture and alignment [17, 40, 43, 47]. No studies evaluating global mobility outside the cervical region have been identified. Similarly to studies on cervical spine posture, it is difficult to assert a complete consensus in studies addressing global posture. On one hand, there are studies suggesting that global posture is affected in individuals with TMD [9, 10], while on the other hand, some studies argue the exact opposite [39, 46]. The discrepancies observed among study results may, similar to cervical spine posture, be influenced by factors such as pain, pain characteristics and the severity of TMD [9, 10]. Studies concluding that global posture is altered in individuals with TMD include these relevant criteria [9, 10], whereas studies reaching the opposite conclusion do not address such criteria or parameters [39, 46], which supports our perspective. The influence of pain and TMD severity on global posture can be explained by a chain reaction process, where both parameters primarily focus on the craniocervical region [37, 45, 47]. The craniocervical postural disturbance caused by forward head posture, characterized by upper cervical extension or lower cervical flexion [6] often accompanying TMD [37, 45, 47], can also negatively impact cervical mobility, considering the relationship between cervical mobility and forward head posture [50, 51]. Since cervical mobility is achieved through the active involvement of the upper, middle and thoracic regions [11], it can be inferred that cervical hypomobility and cervical spine postural disorder may also adversely affect thoracic mobility and posture. Considering the regional interdependence theory by Wainner *et al*. [12], it can be anticipated that postural and mobility impairments in the thoracic region may extend to the lumbar region. However, it should be noted that the primary elements serving as the foundation for linking the chain of events are the muscular and postural maladaptive changes caused by pain and the severity of TMD. Due to the inclusion of individuals with TMD who did not need treatment in our study, the average pain intensity and severity of TMD may not have been at a level that could significantly affect either cervical or global posture, or mobility. As a result, the characteristics of these parameters may have been similar to those of healthy individuals in this study.

The common link that enables the establishment of a relationship between two distinct clinical conditions, such as TMD and cervical hypomobility, is forward head posture. Forward head posture is observed in individuals with TMD with joint and/or muscular pain [37, 45, 47], and it also leads to cervical hypomobility [50]. This makes the postural disorder, serving as a bridge between TMD and cervical hypomobility, more comprehensible and acceptable. In our study, the similarity in craniovertebral angle values between individuals with TMD and healthy individuals may have resulted in both groups having similar cervical spine mobility. Similarly, both groups in the study had mandibular mobility averages above the reference values [52]. This suggests that the inclusion of individuals with TMD that did not need treatment, and consequently had low severity of TMD, may have been a determining factor. The fact that the masticatory muscles and lateral capsule pain threshold measurements in the individuals with TMD were above the threshold values typically associated with myogenic and internal derangement development [48, 53] supports our view.

In our study, pressure pain threshold of the masticatory muscles (except for right anterior temporalis) and lateral capsule in TMD cases were significantly lower compared to the control group. The reduced pressure pain threshold in both the lateral capsule and masticatory muscles in the study group are associated with pathological changes triggered by articular adaptation, which is characterized by posterior-superior translation of the mandibular condyle due to DDwR. The translation of the condyle may have caused compression and inflammation in the bilaminar zone [6, 7], potentially contributing to sensitivity in the lateral capsule. The posterior-superior movement of the mandibular condyle and the reduction of the physiological joint space due to DDwR may have led to excessive activation of the masticatory muscles [54], potentially contributing to the observed sensitivity in these muscles. In our study, although the pressure pain threshold of the masticatory muscles in TMD cases were significantly lower compared to the control group, the threshold values were not severe enough to produce symptomatic effects or prompt individuals to seek treatment. This finding confirms that, despite the adaptive tissue capacity in individuals with DDwR, certain modifications in pain thresholds do develop to some extent [53]. In both groups, including the study group, the pain threshold values of the masticatory muscles exceeded the threshold values of 1.5 kg/cm^2^ and 2.7 kg/cm^2^, which are considered critical for the development of myofascial pain in the masseter and temporalis muscles [48]. Interestingly, while there was a significant difference between the groups in the right upper trapezius muscle, no such difference was observed in the left upper trapezius muscle. The pressure pain threshold of the upper trapezius muscle in the study group was significantly lower compared to healthy individuals. The fact that this difference was observed only in the right upper trapezius may be attributed to the dominant side being the right side in nearly all TMD cases. The observed difference in the right upper trapezius muscle alone may be influenced by the fact that the dominant side was the right side in nearly all individuals with TMD. Pressure pain threshold values on the dominant side are generally lower compared to the non-dominant side [55]. This suggests that the increased activation of the upper trapezius muscle, observed alongside heightened masticatory muscle activity in individuals with TMD, may initially manifest as increased activation in the dominant-side upper trapezius muscle [38]. The increased muscle activation may have contributed to the sensitivity observed in the upper trapezius muscle [6]. Similar to the masticatory muscles, the mean pressure pain threshold values of the upper trapezius muscles in both groups exceeded the threshold of 3.1 kg/cm^2^ [56].

There is already a study examining head posture and the biomechanics of the masticatory muscles. In the related study, it was observed that as head anterior tilt increased, there was a rise in the tone and stiffness of the masticatory muscles (masseter and anterior temporalis). However, this increase was not predominantly significant [56]. As can be inferred from the related study, head anterior tilt has a limited effect on the tone of the masticatory muscles. The similar biomechanical characteristics of the masticatory muscles in our study groups can be explained by their comparable cervical spine posture. The inter-group difference observed only in the left semispinalis muscle’s elasticity may have been influenced by individuals’ habitual daily postures [57]. As also noted in terms of global posture and mobility, the similarity in postural characteristics and mobility of the cervical region in both groups may have led to similar biomechanical properties in the thoracic and lumbar region muscles as well.

The study has several limitations. The first limitation is the non-use of magnetic resonance imaging in the diagnosis of disc displacement. As a result, it has not been fully determined whether disc displacement, which can be observed in asymptomatic healthy individuals, exists. Another limitation is that, although the absence of treatment needs in the individuals within the study group suggests that the severity of TMD or its symptoms is extremely low, the severity of TMD or its symptoms were not evaluated, as the previously reported study [40]. A further limitation is the inclusion of TMD cases that do not in need of treatment in the study. Another limitation is the lack of utilization of DC/TMD Axis II, which includes psychosocial factors and subjective pain experiences, due to the scope of our study. As a result, the multifaceted nature of pain could not be comprehensively assessed in our study.

## 5. Conclusions

This study demonstrated that young adults with DDwR exhibit global posture, mobility and muscle biomechanical characteristics that are largely similar to those of healthy individuals. Additionally, the study revealed that pressure pain threshold, particularly at the joint capsule and in most other measurement points except for a few, was reduced in young adults with temporomandibular disorders.
